# HER2-low prevalence among Hispanic/Latino women with breast cancer: A systematic review and meta-analysis

**DOI:** 10.1371/journal.pone.0315287

**Published:** 2024-12-12

**Authors:** Daniel Felipe Mendivelso-González, Daniel Clavijo Cabezas, Luisa Montoya, Merideidy Plazas Vargas, Patricia López-Correa, Eugenia Colón, Rafael Parra-Medina

**Affiliations:** 1 Department of Pathology, Instituto Nacional de Cancerología, Bogotá, Colombia; 2 Department of Pathology, Fundación Universitaria de Ciencias de la Salud—FUCS, Bogotá, Colombia; 3 Department of Clinical Epidemiology and Biostatistics, Pontificia Universidad Javeriana, Bogotá, Colombia; 4 Department of Epidemiology, Fundación Universitaria de Ciencias de la Salud—FUCS, Bogotá, Colombia; 5 Department of Women’s and Children’s Health, Karolinska Institutet and S:t Göran’s Hospital-Unilabs, Stockholm, Sweden; 6 Research Institute, Fundación Universitaria de Ciencias de la Salud—FUCS, Bogotá, Colombia; University of Wisconsin-Milwaukee Joseph J Zilber School of Public Health, UNITED STATES OF AMERICA

## Abstract

**Purpose:**

HER2-low has garnered significant attention for the treatment of HER2-negative breast cancer. We aimed to determine the prevalence of HER2-low expression in Hispanic/Latino women with breast cancer (BC).

**Methods:**

We searched in Embase, LILACS, and Medline databases for articles reporting the expression of HER2 immunohistochemistry with scores reported as 0, 1+, 2+, or 3+, with equivocal cases (2+) confirmed through in situ hybridization (ISH).

**Results:**

A total of 12 articles were finally included, comprising 73,467 individuals. The prevalence of HER2-zero, HER2-low and HER2 positive cases among all BC (0, 1+, 2+/ISH-, 2+/ISH+ and 3+), was 45.0%, 32.0%, and 23.0%, respectively. The prevalence of HER2-zero and HER2-low expression among negative cases (0, 1+ and 2+/ISH-), was 53.0% and 47.0%, respectively.

**Conclusion:**

There is an important percentage of Hispanic/Latino individuals who would benefit from HER2-targeted therapies, even in HER2 negative cases. Additional research on the prevalence of HER2-low tumors across a wider range of Latin American countries is required to better understand the molecular epidemiology of this biomarker within the Hispanic/Latino population.

## Introduction

In 2022, breast cancer (BC) had the second highest incidence and the fourth highest mortality rate among all cancers worldwide, regardless of gender [1]. Within Latin America, BC ranks second in incidence and mortality, with age-standardized rates reaching 52 and 13.2 per 100,000 individuals, respectively [2]. BC can be classified based on morphology and degree of invasion, with invasive ductal and invasive lobular adenocarcinomas accounting for approximately 80.0% and 8.0% of all BC, respectively [3]. Moreover, the expression of estrogen receptors (ER), progesterone receptors (PR), and the Human Epidermal Growth Factor Receptor 2 (HER2) has established a molecular classification of the disease. Approximately 80.0% of BC tumors are ER and/or PR positive, 23.0% are HER2 overexpressed, and 13.0% are triple-negative (TNBC). These molecular subtypes play a critical role in determining the prognosis and treatment strategies for BC patients, and specific HER2-directed treatments such as trastuzumab and pertuzumab, are being used as first-line therapies to treat HER2-positive tumors [4, 5].

In the American Society of Clinical Oncology/College of American Pathologists (ASCO/CAP) guidelines on HER2 testing [6], immunohistochemistry (IHC) expression is classified as negative for 0 or 1+, equivocal for 2+, and positive for 3+ scores. Equivocal cases should be confirmed by an in-situ hybridization (ISH) technique, which categorizes HER2 2+/ISH- as negative and 2+/ISH+ as positive cases [6]. In recent years, HER2-low, defined as HER2 IHC 1+ or 2+/ ISH-, has emerged as a significant subclassification, sparking numerous molecular and clinical studies [7]. The 2022 DESTINY-Breast04 trial showcased a substantial survival improvement among HER2-low BC patients treated with trastuzumab-deruxtecan, with a progression-free survival (PFS) of 10 months and an overall survival (OS) of 23.4 months for patients treated with this antibody drug conjugate. In contrast, patients treated with other drugs experienced PFS and OS rates of 5 and 16 months, respectively [8].

BC in Hispanic/Latino women, defined as women with Central o South American culture or origin regardless of race, exhibit notable differences compared to non-Hispanic individuals [9, 10]. Some studies have reported that Hispanic/Latino women are more likely to be diagnosed with BC at a younger age and in more advanced stages compared to non-Hispanic White (NHW) individuals [11, 12]. While age-adjusted incidence rates of BC are lower for Hispanic/Latino women than for NHW (93.7 vs. 130.8 per 100,000), the reduction in mortality rates has been less pronounced for Hispanic/Latino women in recent years (29.0% vs. 39.0%) [13, 14]. Furthermore, Hispanic/Latino women have a higher risk of developing HER2 positive (15.6% vs. 12.5%) and TNBC (10.0–18.0% vs. 8.0–15.0%) compared with NHW, which are considered highly aggressive subtypes [15, 16].

Despite the growing recognition of the potential of HER2-low in the treatment of HER2-negative BC, its prevalence among Hispanic/Latino individuals, particularly those from Latin America, remains unknown. Therefore, our systematic review and meta-analysis aim to shed light on the prevalence of HER2-low expression in Hispanic/Latino women with BC, a crucial step in understanding the molecular subtypes and potential treatment strategies for this population.

## Materials and methods

We conducted a systematic review and meta-analysis of studies that reported the prevalence of HER2-low expression in Hispanic/Latino women. The review protocol was developed following the Preferred Reporting Items for Systematic Reviews and Meta-analyses (PRISMA) reporting guidelines. This review is registered in the international PROSPERO database under the code CRD42024542783.

### Inclusion criteria

The review included original studies such as cohort and cross-sectional published up to April 2024, regardless of language. Only studies reporting the prevalence of HER2-low based on IHC, or the expression of HER2 IHC categorized as 0, 1+, 2+, or 3+, with equivocal cases (2+) confirmed through ISH, were considered. Hispanic/Latino women residing in Latin America and abroad were included.

### Exclusion criteria

Exclusion criteria included:

1. Studies with inconsistencies between the text and the data from the tables.
2. Studies that did not differentiate HER2 expression results between Hispanic/Latino and other ethnic groups.
3. Studies that exclusively included HER2-positive cases

### Information sources and search strategy

The search for relevant articles was conducted using the following databases: Embase, LILACS, and Medline. We also retrieved gray literature from Google scholar. We searched for articles published up to April 12, 2024, without any language restrictions. Our search used specific Medical Subject Headings (MeSH) terms such as “Breast Neoplasms”, “Genes, erbB-2”, “Receptor, ErbB-2”, “Immunohistochemistry” and “Hispanic or Latino”. Each search concept was supplemented with pertinent free-text terms, which, when suitable, were truncated and combined with proximity operators for enhanced accuracy (S1 Table in S1 File).

### Study selection

Two primary reviewers (DMG, DCC) independently screened the studies by title and abstract for study selection. Then, the same authors assessed the full text of all screened articles, excluding those not meeting the eligibility criteria. Any disagreements between the authors were resolved by another author (RPM). We also conducted citation chasing, searching for relevant articles within the references of the studies already included in our review. The final selection was based on either the full text of the publication or the abstract of conference presentations. Finally, all data included was reviewed by the authors.

### Data collection process and data extraction

When available, the following data were extracted from each article: year of publication, country, study design, breast cancer subtype, type of HER2 antibody used, HER2 IHC scoring guidelines implemented, number of individuals studied, and HER2 IHC expression levels (reported as 0, 1+, 2+, or 3+), with confirmation of equivocal cases by ISH. Disagreements were resolved by consensus. If the required data were not complete, attempts were made to contact the authors to obtain the missing information.

### Risk of bias and applicability

A checklist based on the Joanna Bridge Institute (JBI) Critical Appraisal Checklist for Systematic Reviews and Research Syntheses [17], was implemented to assess the methodological quality and applicability of the studies. Two authors (DMG, RPM) answered eight questions for cross-sectional studies and eleven questions for cohort studies. Each question was analyzed as ’yes’ (Y), ’no’ (N), ’unclear’ (U) or ’not applicable’ (NA).

### Summary measures

The primary outcomes were the prevalence of HER2-low among all cases of BC (0, 1+, 2+/ISH-, 2+/ISH+, and 3+) and the prevalence of HER2-low among HER2-negative BC cases (0, 1+, and 2+/ISH-).

### Data synthesis and analysis

All quantitative analyses of the studies included in the research were conducted using Stata 17 (StataCorp 2017, Stata Statistical Software, Release 15, College Station, TX, Stata Corp LLC) with the Metaprop package. Two meta-analyses employing a fixed-effect model were used to pool the prevalence of HER2-low among all cases of BC, and the prevalence of HER2-low among HER2-negative cases. We conducted country-specific analyses to examine the differences in prevalence across the countries included in the studies, as each Latin American country has distinct demographic and genetic characteristics. Heterogeneity was estimated using I^2^, with the following cutoff points: a value less than 25.0% indicated low heterogeneity, 25.0% to 75.0% indicated moderate heterogeneity, and greater than 75.0% indicated high heterogeneity. The significance level was set at 5.0%. Heterogeneity was assessed by sample size, country, and study design. Moreover, the clinicopathological characteristics of the tumor samples from each study were collected and categorized based on HER2 expression levels.

### Ethics statement

Ethics approval was not required for this systematic review.

## Results

### Systematic literature review

**Fig 1:**
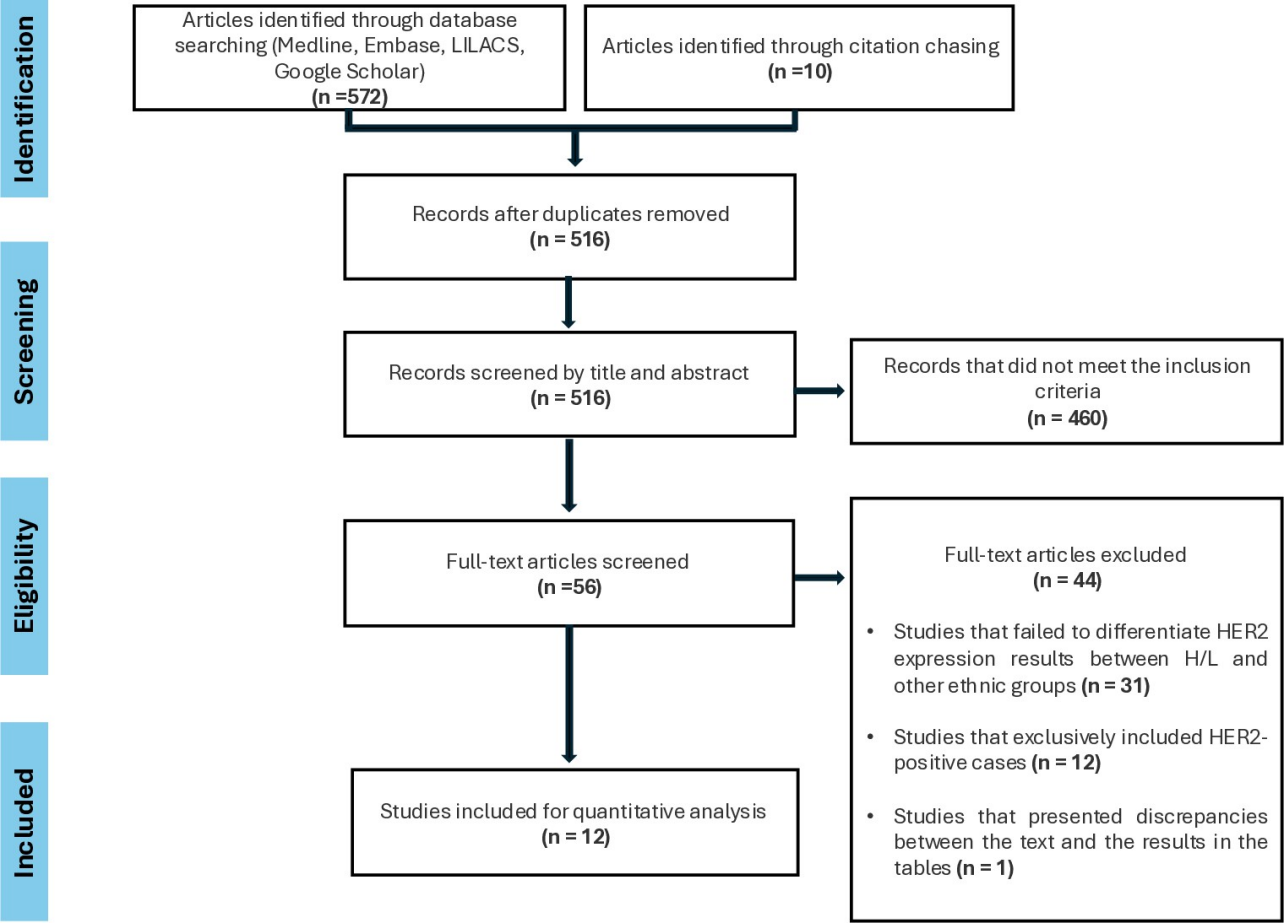
Search strategy.

The PRISMA flow diagram is shown in Fig 1, summarizing the selection process. After removing duplicates, 516 out of 582 citations were screened based on their title and abstract. After screening, 460 articles did not meet the inclusion criteria, and 56 articles were selected for full-text assessment. Forty-four articles were excluded based on the following criteria: 31 studies did not differentiate HER2 expression results between Hispanic/Latinos and other ethnic groups, 12 articles exclusively included HER2-positive cases, and one paper presented discrepancies between the text and the results in the tables (S2 File). Finally, 12 articles were included in the review [18–29] (Table 1). Out of these 12 studies, data were extracted from an abstract in 4 cases [20–22, 25]. The studies included individuals from Brazil (n = 6), Colombia (n = 2), Mexico (n = 2), and Hispanic women living in the United States (US) (n = 2). Two research theses written in Portugues and Spanish, were retrieved from the gray literature search [18, 23]. The review included seven cohort studies and five cross-sectional studies. Seven studies reported the prevalence of HER2-low tumors among all BC (0, 1+, 2+/ISH-, 2+/ISH+, and 3+) [20–23, 27–29], while the other five studies reported the prevalence of HER2-low among HER2-negative cases (0, 1+, and 2+/ISH-) [18, 19, 24–26], with sample sizes ranging from 55 to 3,737 and from 44 to 62,985 individuals, respectively. Three articles reported the antibody used for HER2 evaluation, which was different in each case. Seven articles reported the IHC results using the ASCO/CAP guidelines, with 3 using the 2007 version and 5 using the 2018 update.

**Table 1:** General characteristics of included articles.

									HER2-zero	HER2-low			
Study	Type of study	Country	Types of cancer included	Antibody used		Scoring system	Number of individuals	Mean age	IHC 0	IHC 1+	IHC2+ ISH-	HER2-zero prevalence (%)	HER2-low prevalence (%)
Pinheiro et al. 2024 (18)	Cohort	Brazil	I-III		NA	ASCO/CAP guidelines (2018)	512	51	230	226	56	44.9	55.1
Peiffer et al. 2023 (19)	Cohort	US/ Hispanics	Invasive		NA	NA	62,985	62.4	24,536	27,978	10,471	39.0	61.0
Fernandes et al. 2023 (20)	Cross-sectional	Brazil	Invasive stages I-III		NA	ASCO/CAP guidelines (2018)	179	55	128	51		71.5	28.5
Martinez-Cannon et al. 2023 (21)	Cohort	Mexico	Metastatic		NA	NA	44	51	18	26		40.9	59.1
Vargas et al. 2023 (22)	Cohort	Colombia	stages I-III		NA	NA	422	NA	325	97		77.0	23.0
López-Altamirano et al. 2023 (23)	Cross-sectional	Mexico	All		NA	ASCO/CAP guidelines (2007)	159	48,1	119	40		74.8	25.2
Jiang et al. 2022 (24)	Cohort	US/ Hispanics	Metastatic		NA	ASCO/CAP guidelines (2018)	1682	NA	646	1036		38.4	61.6
Reinert et al. 2021 (25)	Cohort	Brazil	All		NA	NA	331	NA	164	167		49.5	50.4
Moura Leite et al. 2021 (26)	Cohort	Brazil	All		NA	ASCO/CAP guidelines (2007)	855	45	570	181	104	66.7	33.3
Arias et al. 2017 (27)	Cross-sectional	Brazil	Invasive		Anti-HER-2 antibody PATHWAY ® (4B5)	ASCO/CAP guidelines (2007)	1,029	55,4	627	313	89	60.9	39.1
Plata et al. 2013 (28)	Cross-sectional	Colombia	Invasive		Herceptest methodology (Dako K5204)	ASCO/CAP guidelines (2007)	2,709	NA	1145	613	951	42.3	57.7
Wludarski et al 2011 (29)	Cross-sectional	Brazil	Invasive		Antibody SP3 (code RM-9103-S, NeoMarkers, Lab Vision Corp, Fremont, CA)	ASCO/CAP guidelines (2007)	651	NA	209	148	294	32.1	67.9

NA, not available. ASCO/CAP, American Society of Clinical Oncology / College of American Pathologists. IHC, immunohistochemistry. ISH, in situ hybridization

### General clinicopathological information

The twelve studies included a total of 73,467 Hispanic/Latino women with BC [18–29]. Age information was provided in seven articles, revealing a mean age of 51 years. Table 2 displays the available clinicopathological information taken from the included studies.

**Table 2:** Clinicopathological features of included cases.

	Characteristics (number of individuals with available data)		n	%
All BC (HER2 IHC 0, 1+, 2+/ISH-, 2+/ISH+ and 3+)	Histology (n = 2,042)	Ductal carcinoma	1776	87.0
		Lobular carcinoma	136	6.7
		Other	130	6.4
	Lymphovascular invasion (n = 1,487)	Positive	547	36.8
		Negative	940	63,2
	KI-67 expression (n = 1,487)	High	283	19.0
		Low	1204	81.0
HER2-negative tumors (IHC 0, 1+ and 2+/ISH)	Histology (n = 1,542)	Ductal carcinoma	1340	86.9
		Lobular carcinoma	113	7.3
		Other	89	5.8
	Molecular subtypes (n = 1,186)	Luminal	751	63.3
		TNBC	435	36.7
	Clinical stage (n = 950)	I	59	6.2
		II	367	38.6
		III	524	55.2
	Grade (n = 1,366)	1	69	05.0
		2	605	44.3
		3	692	50.7
HER2-low tumors (IHC 1+ and 2+/ISH-)	Histology (n = 655)	Ductal carcinoma	594	90.7
		Lobular carcinoma	31	4.7
		Other	30	4.6
	Molecular subtypes (n = 491)	Luminal	367	74.7
		TNBC	124	25.2
	Clinical stage (n = 607)	I	32	5.3
		II	259	42.7
		III	316	52.1
	Grade (n = 629)	1	45	7.1
		2	299	47.5
		3	285	45.3
	Lymphovascular invasion (n = 333)	Positive	62	18.6
		Negative	271	81.4
	KI-67 expression (n = 366)	High	137	37.5
		Low	229	62.5

BC, breast cancer. IHC, immunohistochemistry. ISH, In situ hybridization. TNBC, triple negative breast cancer

In the seven articles that included HER2-zero, HER2-low, and HER2-positive tumors [20–23, 27–29], histological tumor type data were sourced from four articles [20, 21, 27, 29], revealing a prevalence of 87.1% for ductal carcinoma. Two articles [20, 27], provided insights into lymphovascular invasion and KI-67 expression, with prevalences of 36.8% and 19.0%, respectively.

In the group of five articles that included exclusively HER2 negative cases (0, 1+ and 2+/ISH-) [18, 19, 24–26], data on histological type were available from two articles [18, 26], indicating a prevalence of 86.9% for ductal carcinoma. Two articles [25, 26] provided insights into the molecular subtypes of tumors, with a prevalence of 63.3% for luminal carcinoma. Clinical stage data, sourced from two articles [18, 26], showed a prevalence of 55.2% for stage III. Histological grade information, reported in two articles [18, 26], revealed a prevalence of 50.7% for grade 3.

Specific clinicopathological information on HER2-low tumors was available from five studies [18, 20, 23, 25, 26]. Data on histological type and grade were available from four articles [18, 20, 23, 26], indicating a prevalence of 90.7%. Regarding molecular subtypes of HER2-low cases, insights from three articles [23, 25, 26], revealed a proportion of 74.7% for luminal types. Clinical stage information, sourced from three articles [18, 23, 26], showed a prevalence of 52.1% for stage III. Histological grade data, reported in four articles [18, 20, 23, 26], revealed a prevalence 47.5% for grade 2. Lymphovascular invasion data, available from two articles [18, 20], showed a prevalence of 18.6%. Additionally, KI-67 expression data, sourced from two articles [18, 20], indicated a prevalence of high-expressing tumors at 37.5%.

### HER2-low expression in Hispanic/Latino women

**Fig 2:**
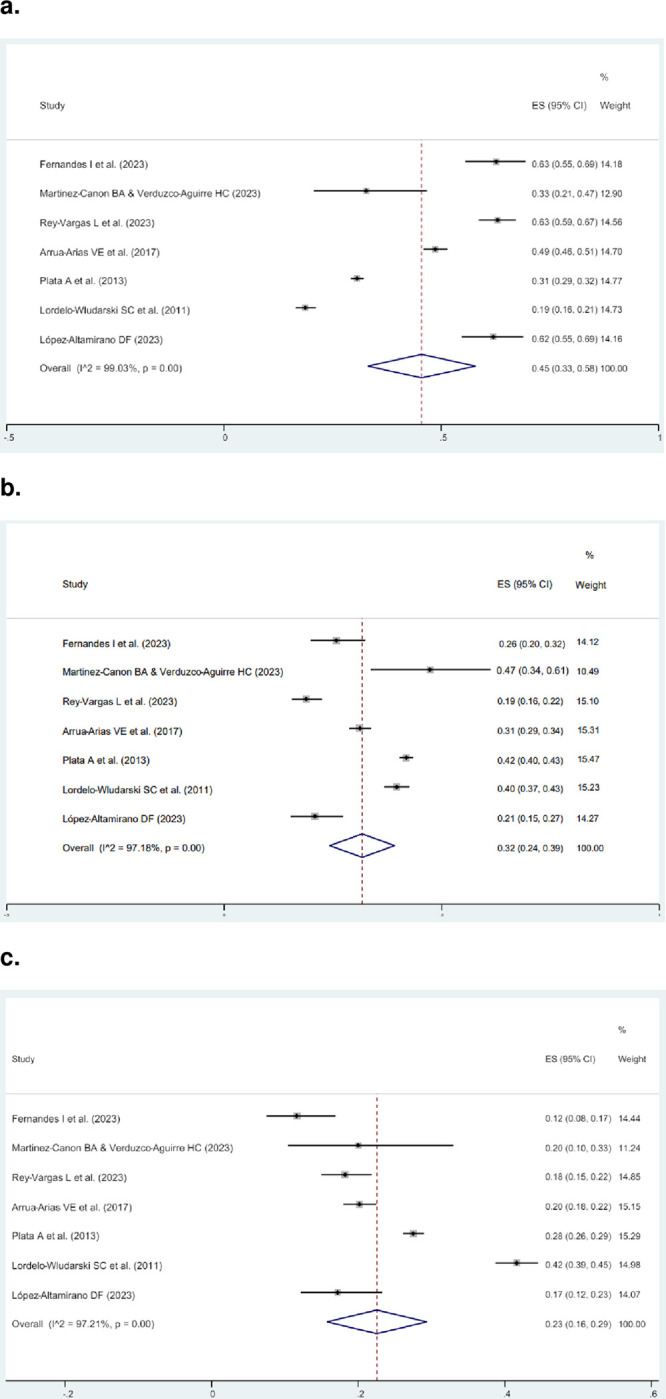
Meta-analysis showing, among all BC cases (HER2 IHC 0, 1+, 2+/ISH-, 2+/ISH+, and 3+), the prevalence of HER2-zero (a), HER2-low (b), and HER2-positive expressing tumors.

Within the seven articles reporting HER2-zero, HER2-low, and HER2-positive cases [20–23, 27–29] (S2 Table in S1 File), with a total of 7,102 individuals from Brazil, Colombia, and Mexico, the prevalence ranged from 18.7% to 63.1% for HER2-zero tumors, from 18.8% to 47.3% for HER2-low tumors, and from 11.6% to 41.6% for HER2-positive tumors. A meta-analysis of these articles was conducted to identify the prevalence of HER2-low among all BC in Hispanic/Latino women. The prevalence of HER2-zero, HER2-low and HER2 positive cases among all BC (0, 1+, 2+/ISH-, 2+/ISH+, and 3+) was 45.0% (95% CI 33.0–58.0%), 32.0% (95% CI 24.0–39.0%), and 23.0% (95% CI 16.0–29.0%), respectively (Fig 2). Country-specific analysis revealed that Colombia exhibited the highest prevalence of HER2-low expression at 38.0% (95% CI 36.0–39.0%), while Mexico had the lowest at 25.0% (95% CI 20.0–30.0%) (S1 Fig in S1 File). Additionally, Colombia showed the highest prevalence of HER2-positive tumors at 26.0% (95% CI 25.0–37.0%), while Mexico had the lowest at 18.0% (95% CI 13.0–23.0%) (S2 Fig in S1 File).

**Fig 3:**
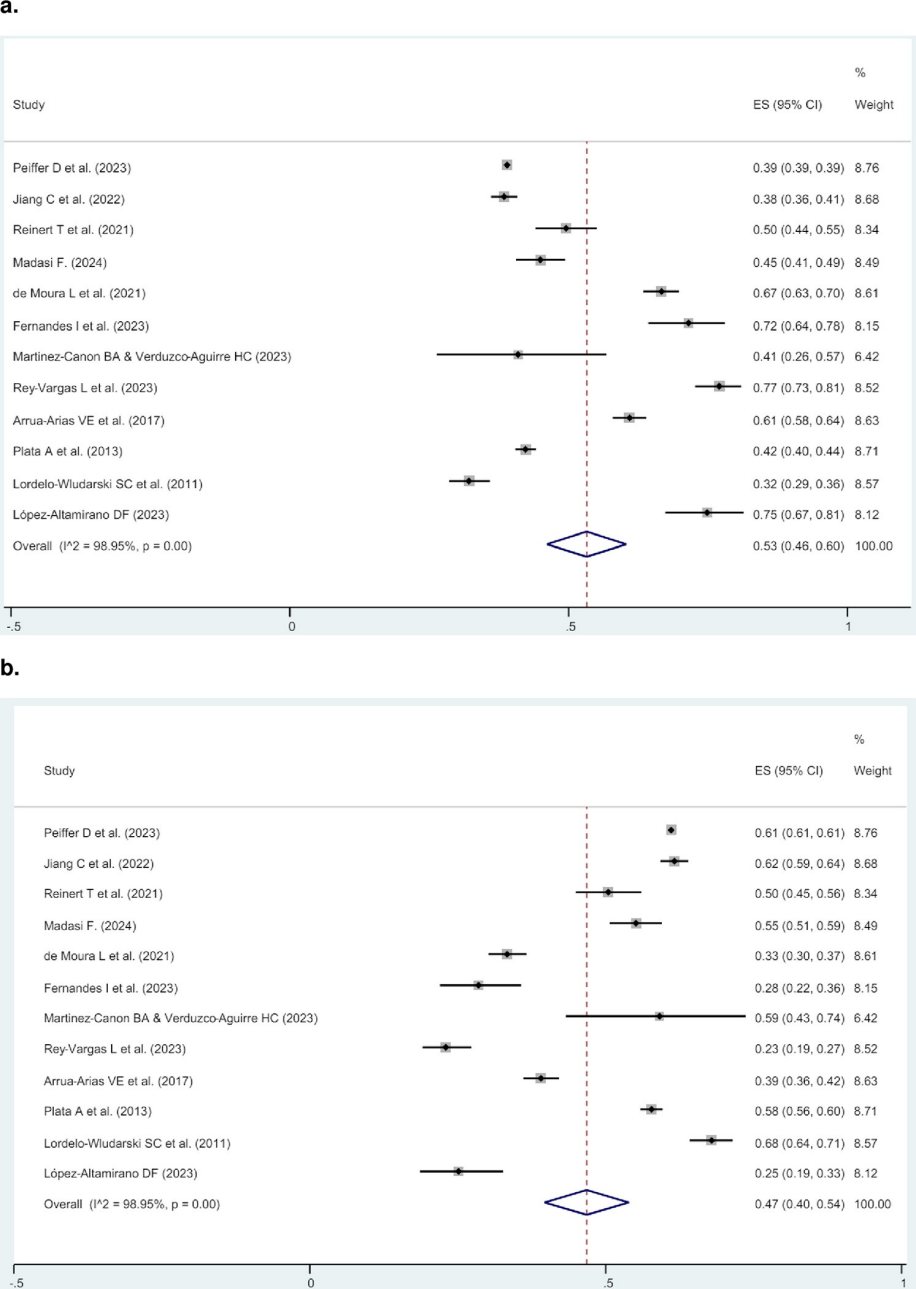
Meta-analysis showing, among HER2-negative cases (IHC 0, 1+, and 2+/ISH), the prevalence of HER2- zero (a), and HER2-low expressing tumors (b).

The cases from these seven articles (excluding HER2-positive tumors) were then combined with individuals from five other studies that only included HER2-zero and HER2-low tumors [18, 19, 24–26]. This resulted in twelve studies with 71,558 individuals from Brazil, Colombia, Mexico, and Hispanic/Latino women living in the US, all providing data on HER2-zero and HER2-low expression (Table 1). In this group, the prevalence values for HER2-zero and HER2-low tumors ranged from 32.1% to 77.0%, and from 23.0% to 67.9%, respectively. A second meta-analysis was conducted to determine the prevalence of HER2-low among HER2-negative cases. The prevalence of HER2-zero and HER2-low among HER2-negative cases (0, 1+, and 2+/ISH-), was 53.0% (95% CI 46.0–60.0%) and 47.0% (95% CI 40.0–54.0%), respectively (Fig 3). Country-specific analysis revealed that Hispanic/Latino living in the United States (US) reported the highest prevalence of HER2-low expression at 61.0% (95% CI 61.0–61.0%), followed by Colombia with 52.0% (95% CI 50.0–53.0%), while Mexico had the lowest at 31.0% (95% CI 25.0–37.0%) (S3 Fig in S1 File).

### Quality assessment

Twelve articles underwent quality evaluation, with the majority meeting the JBI criteria for cohort studies (seven articles) and cross-sectional studies (five articles) (S3 and S4 Tables in S1 File). Among the cohort studies, Reinert et al. [25] did not provide information on follow-up time, and two articles did not include confounding factors in their analyses [18, 25]. Conversely, four studies must have specified the guidelines for interpreting HER2 IHC results [19, 21, 22, 25]. In the cross-sectional studies group, four articles did not assess confounding factors [19, 21, 22, 25], and only three articles specified the antibody used to assess HER2 expression [27–29].

## Discussion

Despite the essential role of tumor characterization in cancer management, there is a significant gap in molecular data for Hispanic/Latino populations, especially in Latin American countries. This systematic review and meta-analysis found that the prevalence in Hispanic/Latino women of HER2-zero, HER2-low and HER2 positive cases among all BC (0, 1+, 2+/ISH-, 2+/ISH+, and 3+) was 45.0% (95% CI 33.0–58.0%), 32.0% (95% CI 24.0–39.0%), and 23.0% (95% CI 16.0–29.0%), respectively. This proportion seems to vary significantly across different regions.

Horisawa et al. [30] found among 4,918 Japanese women with BC, the prevalence of HER2-low was 64.4% (17.1% HER2-positive, 18.5% HER2-zero). In Germany, Denkert et al. [31] reported a HER2-low prevalence of 31.0% among 3,512 women (34.0% HER2-positive, 35.0% HER2-zero). Similarly, Gampenrieder et al. [32] found that among 1,729 BC cases from Austria, 35.2% were HER2-low (20.3% HER2-positive, 44.5% HER2-zero). In the US, Zhang et al. [33] discovered a prevalence of HER2-low cases of 31.0% (10.6% HER2-positive, 58.4% HER2-zero) among 281 individuals.

Conversely, the prevalence of HER2-low and HER2-zero expression among HER2-negative cases (0, 1+, and 2+/ISH-), was 47.0% (95% CI 40.0–54.0%) and 53.0% (95% CI 46.0–60.0%), respectively. Several meta-analyses have reported the prevalence of HER2-low expression among HER2-negative tumors, with most of the data coming from North America, Asia, and Europe: Yang et al. [34] found a prevalence of 43.02% for HER2-low tumors among 78,984 HER2 negative BC; Ergun et al. [35] found that the proportion of HER2-low cases was 65% among 636,535 individuals; Tang et al. [36] analyzed 677,248 cases finding that the prevalence of HER2-low was 65.91%; 1’697,079 tumors were analyzed in the study of Molinelli et al. [37], finding a prevalence of 65.9% of HER2-low cases. It is worth noting that none of these studies included a significant representation of Hispanic/Latino populations. The elevated proportion of HER2-zero cases in Hispanic/Latino individuals compared to other populations may be associated with the higher prevalence of TNBC reported in this population [38]. Martinez et al. [39] analyzed 129,488 BC cases, finding that Hispanic/Latino individuals had a higher likelihood of developing TNBC compared to NHW in the US. This finding is consistent with the results of Hines et al. [40], who found among 3,441 individuals a significantly higher proportion of TNBC tumors in Hispanic/Latino women compared to NHW in the US.

Overall, the prevalence of HER2-low tumors among HER2-negative cases (0, 1+, and 2+/ISH-) is lower in Hispanic/Latino compared to other populations. The study of Peiffer et al. [19] showed that even though Hispanic/Latino individuals had the lowest HER2-low prevalence among other ethnicities in the US, they still have a higher proportion of HER2-low cases compared to Hispanic/Latino individuals residing in Latin American countries, with 61.0% vs. 52.0% (highest HER2-low prevalence found in Latin America). This difference could be partly explained by the higher proportion of HER2-positive cases observed in Hispanic/Latino women. In this review, the prevalence of HER2-positive tumors in Hispanic/Latino was 23.0%, which is different from the proportions observed in three US studies, where the HER2-positive prevalence was 10.6%, 14.9%, and 12.6%, respectively [33, 41, 42].

The higher proportion of HER2-positive BC in Hispanic/Latino may be regulated by the unique genetic admixture seen in Latin American countries [43, 44]. Serrano-Gomez et al. performed a whole-transcriptome and RNA-seq of Colombian BC tumors and estimated their genetic ancestry, finding that Hispanic/Latino women with higher Indigenous American (IA) ancestry fraction, are more likely to develop HER2-positive BC [45]. Furthermore, Marker et al. [46] estimated the genetic ancestry of 1,928 BC tumors from Hispanic/Latino women residing in Peru, Mexico, and Colombia, finding that the likelihood of having an HER2-positive tumor grew by a factor of 1.20 for every 10.0% increase in the proportion of IA ancestry. Research indicates that not only do non-Hispanic Black individuals and non-Hispanic White individuals have a lower proportion of AI ancestry compared to Hispanic/Latino individuals, but Hispanic/Latino individuals living in the US also have a smaller fraction of AI ancestry than those residing in Latin American countries [47, 48].

Some clinicopathological features were associated with HER2-low expression in the included articles. Vargas et al. [22], found that HER2-low was associated with age >50, better-differentiated tumors, lower proliferation index and higher ER expression, compared to HER2 positive cases. Moreover, López Altamirano et al. [23], reported that in HER2-low tumors, High ER and PR expression was associated with PD-L1 negativity while high Ki-67 expression was associated with PD-L1 positivity. Additionally, Pinheiro et al. [18], reported that HER2-low expression was associated with lymphovascular invasion, when compared to HER2-zero cases. The prognosis associations were mixed. Three articles reported a better OS of HER2-low compared to HER2-zero cases, with Peiffer et al [19] finding a better OS for stage III and stage IV BC, and Martinez-Cannon et al. [21] finding a better OS when positive for hormone receptors (HR) or if ≥2 lines of treatment received. In contrast, López-Altamirano et al. [23] found a worse RFS of HER2-low tumors compared to HER2-zero ones if no neoadjuvant therapy was received. Nonetheless, Moura Leite et al. [26] discovered that HER2-low expression had no significant association with OS or RFS compared to HER2-zero expression. On the other hand, two articles found a lower pathological complete response (pCR) to therapy in HER2-low tumors than HER2-zero tumors [19, 25].

Some clinicopathological features were associated with HER2-low expression in the included articles. Vargas et al. [22], found that HER2-low was associated with age >50, better-differentiated tumors, lower proliferation index and higher ER expression, compared to HER2 positive cases. Moreover, López Altamirano et al. [23], reported that in HER2-low tumors, High ER and PR expression was associated with PD-L1 negativity while high Ki-67 expression was associated with PD-L1 positivity. Additionally, Pinheiro et al. [18], reported that HER2-low expression was associated with lymphovascular invasion. The prognosis associations were mixed. Three articles reported a better OS of HER2-low compared to HER2-zero cases, with Peiffer et al. [19] finding a better OS for stage III and stage IV BC, and Martinez-Cannon et al. [21] finding a better OS when positive for hormone receptors (HR) or if ≥2 lines of treatment received. In contrast, López-Altamirano et al. [23] found a worse RFS of HER2-low tumors compared to HER2-zero ones if no neoadjuvant therapy was received. Nonetheless, Moura Leite et al. [26] discovered that HER2-low expression had no significant association with OS or RFS compared to HER2-zero expression. On the other hand, two articles found a lower pathological complete response (pCR) to therapy in HER2-low tumors than HER2-zero tumors [19, 25].

This review is the first of its kind, providing an estimate of the prevalence of HER2-low expression in the Hispanic/Latino population. However, our study has several limitations. The included studies varied in terms of the size of their study populations, and the criteria they used for selecting the cases, indicating a high degree of heterogeneity. In addition, only twelve articles met the inclusion criteria, reflecting limited research on HER2-low expression in Hispanic/Latino populations. This scarcity makes it difficult to generalize these findings to other Latin American countries or to Hispanic/Latino individuals living in non-Latin American regions. Several excluded articles presented valuable information but lacked detailed data on HER2 IHC scoring, merely categorizing cases as "positive" or "negative". Other studies included Hispanic/Latino subjects but did not distinguish their data from those of other ethnicities. On the other hand, the assessment of HER2 can be inconsistent, with variability observed not only among individual pathologists but also across different laboratories. This could be partly explained by the fact that prior to the 2022 DESTINY-Breast04 trial, pathologists were not used to differentiate between 0 and 1+ scores, as both were considered negative with no clinical implications. This may also reflect an information bias, as the measurement and classification of breast cancer could vary between studies published before and after the trial. A key limitation is the variability in the definition of Hispanic/Latino individuals, with differences across sources like the US Census Bureau [49]. The Census definition includes individuals who identify as "Mexican, Mexican American, Chicano," "Puerto Rican," "Cuban," or as being of "another Hispanic, Latino, or Spanish origin" This inconsistency may further contribute to the heterogeneity observed across studies. Lastly only three of the included articles indicated which HER2 antibody was used for assessment, and four articles did not specify which HER2 scoring guidelines they followed. Moreover, not all the articles specified the guidelines used for HER2 IHC scoring, and those that did, referenced guidelines from different years, potentially leading to variations in the reported HER2 scores.

There is significant potential for improvement in obtaining a more accurate estimate of HER2 prevalence among the Hispanic/Latino population. It is crucial to prioritize the publication of data from health centers across countries that currently lack this information. Moreover, improving existing data sources is essential by standardizing data collection processes and including key variables such as genetic ancestry and/or self-identified race. Efforts should also focus on ensuring the inclusion of diverse populations representing the various ethnic groups within each country. It is equally important to report methodological details such as the staining protocols and the antibody that was implemented. Laboratories should strive to adhere to standardized guidelines for HER2 IHC scoring. Finally, recognizing the emerging importance of differentiating HER2-zero from HER2-low should be a priority among laboratories and pathologists.

## Conclusions

This study found that among the Hispanic and Latino population, the prevalence of HER2- low was 32.0% among all breast cancer cases and 47.0% among HER2-negative cases. These percentages are lower than what has been reported in other regions. The difference might be due to the higher IA ancestry fraction found in Hispanic and Latino individuals, which has been linked to a higher proportion of HER2-positive tumors. Additionally, the high incidence of TNBC among Hispanic and Latino individuals might also contribute to the larger proportion of HER2-zero cases observed in this study. However, the limited representation of other Latin American countries makes it difficult to generalize these findings to the Hispanic/Latino community, and the percentages provided should be regarded as approximations. Scoring variability among pathologists and laboratories poses challenges for assessing the prevalence of IHC markers, and implementing digital pathology in laboratories could help overcome these obstacles. Further studies from a broader range of Latin American countries are needed to obtain a more accurate estimate of HER2-low expression prevalence among the Hispanic/Latino population.

## Supporting information

S1 Checklist (DOCX)

S1 File Search strategy, participant selection criteria, HER2 expression prevalence by country, and quality assessment of included studies. (DOCX)

S2 File Articles identified for full text assessment and all data extracted. (XLSX)
