# Supplementary material for: HER2-low prevalence among Hispanic/Latino women with breast cancer: A systematic review and meta-analysis
Source: PLoS One. 2024 Dec 12;19(12):e0315287. doi: 10.1371/journal.pone.0315287 (PMC11637277; doi:10.1371/journal.pone.0315287)
Supplement: S1 File — (DOCX) [file pone.0315287.s002.docx]

**Supplementary material 1**

| **Medline** | ("Breast Neoplasm*"[tiab] OR "Breast Tumor*"[tiab] OR "Breast Cancer*"[tiab] OR "Mammary Cancer*"[tiab] OR "Cancer Mammary"[tiab] OR "Malignant Neoplas* of Breast*"[tiab] OR "Breast Malignant Neoplas*"[tiab] OR "Malignant Tumor of the Breast*"[tiab] OR "Cancer of the Breast*"[tiab] OR "Human Mammary Carcinoma*"[tiab] OR "Human Mammary Neoplas*"[tiab] OR "Breast Carcinoma*"[tiab]) AND (HER2*[tiab] OR HER-2*[tiab] OR ERBB2[tiab] OR ERBB-2[tiab] OR ERB2[tiab] OR ERB-2[tiab] OR "human epidermal growth factor receptor 2"[tiab] OR "ERB receptor 2"[tiab] OR "ERBB receptor 2"[tiab] OR "human epidermal growth factor receptor-2"[tiab] OR "ERB receptor-2"[tiab] OR "ERBB receptor-2"[tiab] OR "erythroblastic oncogene B 2"[tiab] OR "ErbB-2 Receptor"[tiab] OR "CD340 Antigen"[tiab] OR "Erb b2 Receptor Tyrosine Kinases"[tiab] OR "c erbB 2 Protein"[tiab] OR "erbB 2 Proto Oncogene Protein"[tiab] OR "neu Proto-Oncogene Protein"[tiab] OR "Neu Receptor*") AND (("Latin American*" OR Latin-American*) OR Hispanic* OR latin* OR latino* OR (mexico) OR (argentina) OR (bolivia) OR (brasil OR brazil) OR (chile) OR colombia OR (ecuador) OR (guiana OR guyana) OR (guiana francesca OR French guiana OR Guyana francesa) OR (paraguay) OR (peru) OR (suriname) OR (uruguay) OR (venezuela) OR (“antiga e barbuda” OR (antigua AND barbuda) OR “lantigua y barbuda”) OR (bahamas) OR (barbados) OR (belize) OR (“costa rica”) OR (cuba) OR (dominica) OR (“el salvador”) OR (granada OR grenada) OR (guatemala) OR (haiti) OR (honduras) OR (jamaica) OR (nicaragua) OR (panama) OR (“dominican republic” OR “republica dominicana”) OR (santalucia OR santalucia OR saint lucia) OR (“saocristovao e nevis” OR “san kittsnevis” OR (“saint kitts” AND nevis)) OR (“saovicente de grenadines” OR “san vicente y las grenadines” OR (“saint vincent” AND “the grenadines”)) OR (“trinidad et tobago” OR “trinidad y tabago” OR (trinidad AND tobago)) OR (“Puerto rico” OR portorico) OR (guadeloupe OR guadalupe) OR (martinique OR martini) OR (saint martin) OR saint barthelemy) OR ("america latina") AND (Immunohistochemistry OR IHC OR Hybridization OR FISH OR ISH OR DISH OR CISH) |
| --- | --- |
| **Embase** | ('breast neoplasm*':ti,ab,kw OR 'breast tumor*':ti,ab,kw OR 'breast cancer*':ti,ab,kw OR 'mammary cancer*':ti,ab,kw OR 'cancer mammary':ti,ab,kw OR 'malignant neoplas* of breast*':ti,ab,kw OR 'breast malignant neoplas*':ti,ab,kw OR 'malignant tumor of the breast*':ti,ab,kw OR 'cancer of the breast*':ti,ab,kw OR 'human mammary carcinoma*':ti,ab,kw OR 'human mammary neoplas*':ti,ab,kw OR 'breast carcinoma*':ti,ab,kw) AND ('her2*':ti,ab,kw OR 'her-2*':ti,ab,kw OR 'erbb2':ti,ab,kw OR 'erbb-2':ti,ab,kw OR 'erb2':ti,ab,kw OR 'erb-2':ti,ab,kw OR 'human epidermal growth factor receptor 2':ti,ab,kw OR 'erb receptor 2':ti,ab,kw OR 'erbb receptor 2':ti,ab,kw OR 'human epidermal growth factor receptor-2':ti,ab,kw OR 'erb receptor-2':ti,ab,kw OR 'erbb receptor-2':ti,ab,kw OR 'erythroblastic oncogene b 2':ti,ab,kw OR 'erbb-2 receptor':ti,ab,kw OR 'cd340 antigen':ti,ab,kw OR 'erb b2 receptor tyrosine kinase*':ti,ab,kw OR 'c erbb 2 protein':ti,ab,kw OR 'erbb 2 proto oncogene protein':ti,ab,kw OR 'neu proto-oncogene protein':ti,ab,kw OR 'neu receptor*':ti,ab,kw)  AND (('latin american*':ti,ab,kw OR 'latin-american*':ti,ab,kw) OR 'hispanic*':ti,ab,kw OR 'latin*':ti,ab,kw OR ('mexico':ti,ab,kw) OR ('argentina':ti,ab,kw) OR ('bolivia':ti,ab,kw) OR ('brasil':ti,ab,kw OR 'brazil':ti,ab,kw) OR ('chile':ti,ab,kw) OR 'colombia':ti,ab,kw OR ('ecuador':ti,ab,kw) OR ('guiana':ti,ab,kw OR 'guyana':ti,ab,kw) OR ('guiana francesca':ti,ab,kw OR 'french guiana':ti,ab,kw OR 'guyana francesa':ti,ab,kw) OR ('paraguay':ti,ab,kw) OR ('peru':ti,ab,kw) OR ('suriname':ti,ab,kw) OR ('uruguay':ti,ab,kw) OR ('venezuela':ti,ab,kw) OR ('antiga e barbuda':ti,ab,kw OR ('antigua':ti,ab,kw AND 'barbuda':ti,ab,kw) OR 'lantigua y barbuda':ti,ab,kw) OR ('bahamas':ti,ab,kw) OR ('barbados':ti,ab,kw) OR ('belize':ti,ab,kw) OR ('costa rica':ti,ab,kw) OR ('cuba':ti,ab,kw) OR ('dominica':ti,ab,kw) OR ('el salvador':ti,ab,kw) OR ('granada':ti,ab,kw OR 'grenada':ti,ab,kw) OR ('guatemala':ti,ab,kw) OR ('haiti':ti,ab,kw) OR ('honduras':ti,ab,kw) OR ('jamaica':ti,ab,kw) OR ('nicaragua':ti,ab,kw) OR ('panama':ti,ab,kw) OR ('dominican republic':ti,ab,kw OR 'republica dominicana':ti,ab,kw) OR ('santalucia':ti,ab,kw OR 'santalucia':ti,ab,kw OR 'saint lucia':ti,ab,kw) OR ('saocristovao e nevis':ti,ab,kw OR 'san kittsnevis':ti,ab,kw OR ('saint kitts':ti,ab,kw AND 'nevis':ti,ab,kw)) OR ('saovicente de grenadines':ti,ab,kw OR 'san vicente y las grenadines':ti,ab,kw OR ('saint vincent':ti,ab,kw AND 'the grenadines':ti,ab,kw)) OR ('trinidad et tobago':ti,ab,kw OR 'trinidad y tabago':ti,ab,kw OR ('trinidad':ti,ab,kw AND 'tobago':ti,ab,kw)) OR ('puerto rico':ti,ab,kw OR 'portorico':ti,ab,kw) OR ('guadeloupe':ti,ab,kw OR 'guadalupe':ti,ab,kw) OR ('martinique':ti,ab,kw OR 'martini':ti,ab,kw) OR ('saint martin':ti,ab,kw) OR 'saint barthelemy':ti,ab,kw) OR ('america latina':ti,ab,kw) AND (Immunohistochemistry OR IHC OR Hybridization OR FISH OR ISH OR DISH OR CISH) |
| **LILACS** | (("Breast Neoplasm*" OR "Neoplasm Breast*" OR "Breast Tumor*" OR "Tumor Breast*" OR "Breast Cancer*" OR "Cancer Breast*" OR "Mammary Cancer*" OR "Cancer Mammary" OR "Malignant Neoplasm of Breast*" OR "Breast Malignant Neoplasm*" OR "Malignant Tumor of Breast*" OR "Breast Malignant Tumor*" OR "Cancer of Breast*" OR "Cancer of the Breast*" OR "Mammary Carcinoma Human" OR "Carcinoma Human Mammary" OR "Human Mammary Carcinoma*" OR "Mammary Neoplasm Human" OR "Human Mammary Neoplasm*" OR "Neoplasm Human Mammary" OR "Breast Carcinoma*" OR "Carcinoma Breast*")) AND ((her2* OR her-2* OR erbb2 OR erbb-2 OR erb2 OR erb-2 OR "human epidermal growth factor receptor 2" OR "ERB receptor 2" OR "ERBB receptor 2" OR "human epidermal growth factor receptor-2" OR "ERB receptor-2" OR "ERBB receptor-2" OR "erythroblastic oncogene B 2") ) AND ((("Latin American*" OR latin-american* OR hispanic* OR latina* OR latino*) OR (mexico) OR (argentina) OR (bolivia) OR (brasil OR brazil) OR (chile) OR colombia OR (ecuador) OR (guiana OR guyana) OR (guiana francesca OR french guiana OR guyana francesa) OR (paraguay) OR (peru) OR (suriname) OR (uruguay) OR (venezuela) OR (“antiga e barbuda” OR (antigua AND barbuda) OR “lantigua y barbuda”) OR (bahamas) OR (barbados) OR (belize) OR (“costa rica”) OR (cuba) OR (dominica) OR (“el salvador”) OR (granada OR grenada) OR (guatemala) OR (haiti) OR (honduras) OR (jamaica) OR (nicaragua) OR (panama) OR (“dominican republic” OR “republica dominicana”) OR (santalucia OR santalucia OR saint lucia) OR (“saocristovao e nevis” OR “san kittsnevis” OR (“saint kitts” AND nevis)) OR (“saovicente de grenadines” OR “san vicente y las grenadines” OR (“saint vincent” AND “the grenadines”)) OR (“trinidad et tobago” OR “trinidad y tabago” OR (trinidad AND tobago)) OR (“puerto rico” OR portorico) OR (guadeloupe OR guadalupe) OR (martinique OR martini) OR (saint martin) OR saint barthelemy) OR ("america latina")) |

**Supplementary table 1** Search strategy

| Study | Selection criteria for participants |
| --- | --- |
| Pinheiro et al. 2024 (16) | Triple-negative breast cancer cases that underwent surgery and chemotherapy at a Brazilian institution between 2010 and 2016. |
| Peiffer et al. 2023 (17) | Invasive breast cancer (2010-2019), whose cancer was not classified as ERBB2-positive and had ERBB2 IHC results available. |
| Fernandes et al. 2023 (18) | Brazilian center (2021–2022). Included were stage I–III invasive breast cancer cases with data on TILs, HER2, ER, PR, and ISH. Patients who had undergone neoadjuvant therapy were excluded. |
| Martinez-Cannon et al. 2023 (19) | Metastatic breast cancer (2017-2020), including only those with known HER2 status and available survival data. |
| Vargas et al. 2023 (20) | Breast cancer patients from various health institutions in Colombia, diagnosed at clinical stages I to III. |
| López-Altamirano et al. 2023 (21) | Breast cancer diagnosis (2015-2020) and available histopathological information. |
| Jiang et al. 2022 (22) | (2010-2017) Breast cancer cases with complete data on age, survival status, TNM stage 4, hormone receptor status, HER2 expression, and receipt of systemic treatment. Exclusions were HER2−IHC3+, IHC2+/ISH positive, undetermined tumors, or missing survival data. |
| Reinert et al. 2021 (23) | HER2-negative breast cancer treated with neoadjuvant chemotherapy at four institutions in Brazil, using anthracycline- and taxane-based chemotherapy. |
| Moura Leite et al. 2021 (24) | Breast cancer patients treated with neoadjuvant (2007-2018) from Brazil. Eligible patients were aged ≥ 18, had histologically confirmed invasive breast carcinoma at clinical stage I–III, and underwent curative surgery. Exclusions included HER2-positive breast cancer, insufficient tumor data, previous breast cancer, or other synchronous invasive cancers. |
| Arias et al. 2017 (25) | The study investigated the frequency of HER2-positive breast cancer in Brazilian women with invasive breast cancer (2011-2012). |
| Plata et al. 2013 (26) | Invasive breast cancer cases referred to the Department of Pathology at a Colombian Center (2004-2010) to assess HER2 oncogene. |
| Wludarski et al 2011 (27) | Invasive breast carcinoma (2008-2009) with HER2 testing, from different geographic regions of Brazil. |

**Supplementary table 2.** Selection criteria for participants in each study.

|  |  |  | HER2-zero | HER2-low | | HER2-positive | |  |  |  |
| --- | --- | --- | --- | --- | --- | --- | --- | --- | --- | --- |
| Study | Year | Number of individuals | 0 | 1+ | 2+ ISH (-) | 2+ ISH (+) | 3+ | HER2-zero prevalence (%) | HER2-low (1+, 2+ ISH-) prevalence (%) | HER2 positive (3+, 2+ ISH+) prevalence (%) |
| Fernandes et al. 2023 (9) | 2023 | 198 | 128 | 51 | | 23 | | 62.6 | 25.7 | 11.6 |
| Martinez-Cannon et al. 2023 (10) | 2023 | 55 | 18 | 26 | | 11 | | 32.7 | 47.3 | 20.1 |
| Vargas et al. 2023 (11) | 2023 | 516 | 325 | 97 | | 94 | | 63.0 | 18.8 | 18.2 |
| López Altamirano et al. 2023 (12) | 2023 | 192 | 119 | 40 | | 33 | | 62.0 | 20.8 | 17.2 |
| Arias et al. 2017 (16) | 2017 | 1289 | 627 | 313 | 89 | 19 | 241 | 48.6 | 31.2 | 20.2 |
| Plata et al. 2013 (17) | 2013 | 3737 | 1145 | 613 | 951 | 551 | 477 | 30.6 | 41.8 | 27.5 |
| Wludarski et al 2011 (18) | 2011 | 1115 | 209 | 148 | 294 | 127 | 337 | 18.7 | 39.6 | 41.6 |

**Supplementary table 3** Articles reporting HER2-zero, HER2-low, and HER2-positive expression

**
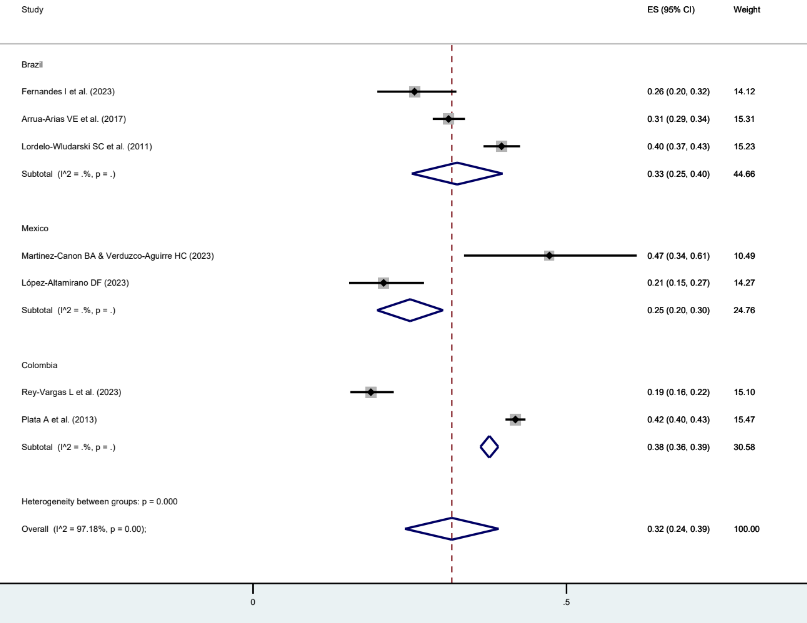
**

**Supplementary Fig. 1** HER2-low prevalence by country among the 7 articles reporting HER2-zero, HER2-low, and HER2-positive expression

**
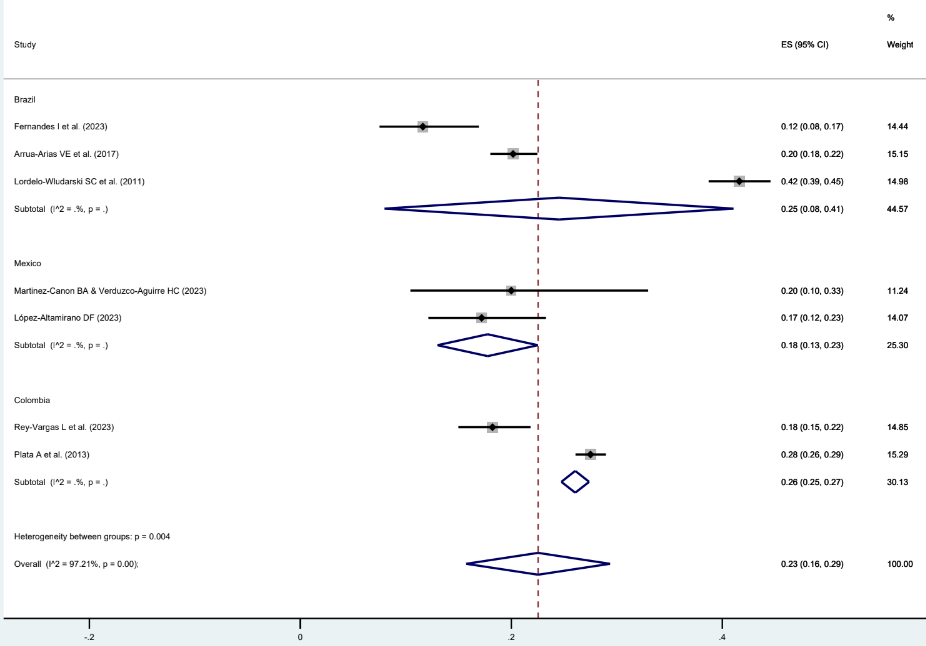
**

**Supplementary Fig. 2** HER2 positive prevalence by country among the 7 articles reporting HER2-zero, HER2-low, and HER2-positive expression

**
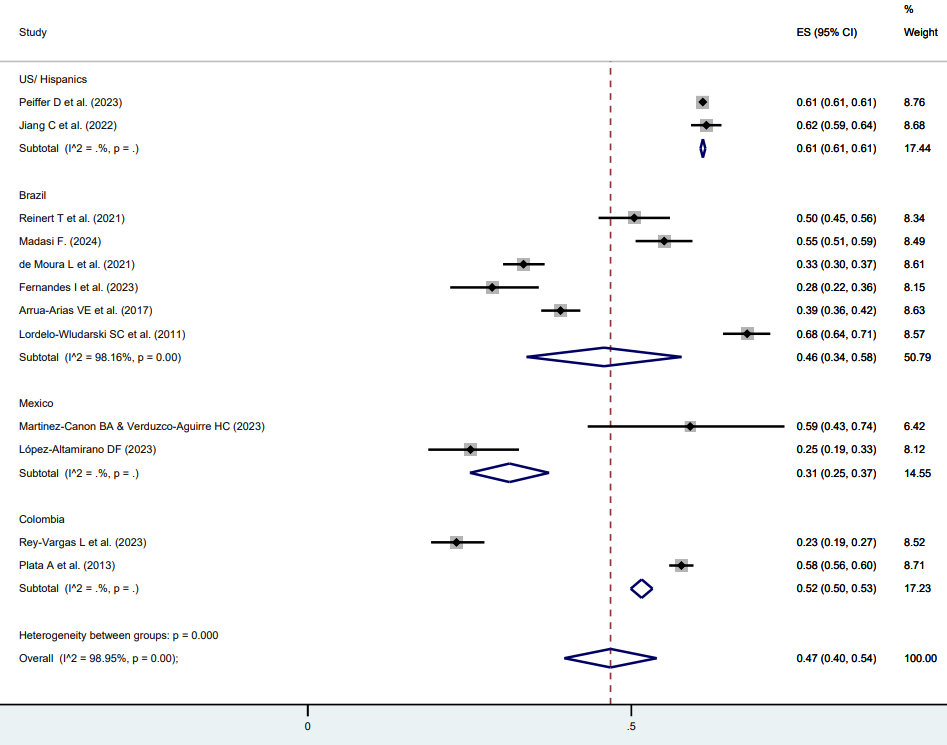
**

**Supplementary Fig. 3** HER2-low prevalence by country, among the 12 articles reporting HER2-zero and HER2-low expression

**a.**

| Ref. | Year | Type of study | Were the two groups similar and recruited from the same population? | Were the exposures measured similarly to assign people to both exposed and unexposed groups? | Was the exposure measured in a valid and reliable way? | Were confounding factors identified? | Were strategies to deal with confounding factors stated? | Were the groups/participants free of the outcome at the start of the study (or at the moment of exposure)? | Were the outcomes measured in a valid and reliable way? | Was the follow up time reported and sufficient to be long enough for outcomes to occur? | Was follow up complete, and if not, were the reasons to loss to follow up described and explored? | Were strategies to address incomplete follow up utilized? | Was appropriate statistical analysis used? | Overall appraisal |
| --- | --- | --- | --- | --- | --- | --- | --- | --- | --- | --- | --- | --- | --- | --- |
| Prognóstico e Características Clínico Patológicas do Câncer de Mama  HER2-Low com Receptores Hormonais Negativos | 2024 | Cohort | Y | Y | Y | N | N | Y | Y | Y | U | N | Y | INCLUDE |
| Clinicopathologic Characteristics and Prognosis of ERBB2-Low Breast Cancer Among Patients in the National Cancer Database | 2023 | Cohort | Y | Y | Y | Y | Y | Y | Y | Y | Y | U | Y | INCLUDE |
| Frequency and prognosis of HER2-low status in Mexican patients with metastatic breast cancer | 2023 | Cohort | Y | Y | Y | Y | Y | Y | Y | Y | N | N | Y | INCLUDE |
| Characterization of HER2-low breast tumors among a cohort of Colombian women | 2023 | Cohort | Y | Y | Y | Y | Y | Y | Y | Y | U | U | Y | INCLUDE |
| Clinical outcomes of de novo metastatic HER2-low breast cancer: a National Cancer Database Analysis | 2022 | Cohort | Y | Y | Y | Y | Y | Y | Y | Y | Y | U | Y | INCLUDE |
| Prevalence of HER2-low and HER2-zero subgroups and correlation with response to neoadjuvant chemotherapy (NACT) in patients with HER2-negative breast cancer | 2021 | Cohort | Y | Y | Y | N | N | Y | Y | Y | U | U | Y | INCLUDE |
| HER2‑low status and response to neoadjuvant chemotherapy in HER2 negative early breast cancer | 2021 | Cohort | Y | Y | Y | Y | Y | Y | Y | Y | U | U | Y | INCLUDE |

**b.**

| Ref. | Year | Type of study | Were the criteria for inclusion in the sample clearly defined? | Were the study subjects and the setting described in detail? | Was the exposure measured in a valid and reliable way? | Were objective, standard criteria used for measurement of the condition? | Were confounding factors identified? | Were strategies to deal with confounding factors stated? | Were the outcomes measured in a valid and reliable way? | Was appropriate statistical analysis used? | Overall appraisal |
| --- | --- | --- | --- | --- | --- | --- | --- | --- | --- | --- | --- |
| Tumor Inltrating Lymphocytes in initial HER2-Low breast cancer | 2023 | Cross-sectional | Y | Y | Y | Y | N | N | Y | Y | INCLUDE |
| Evaluación de la expresión de PD-L1 (sp142) en tejido tumoral en pacientes con cáncer de mama HER2-low y su relación con factores clínico-patológicos: un estudio de corte transversal | 2023 | Cross-sectional | Y | Y | Y | Y | Y | Y | Y | Y | INCLUDE |
| Assessment of HER-2 status in invasive breast cancer in Brazil | 2017 | Cross-sectional | Y | Y | Y | N | N | N | Y | Y | INCLUDE |
| Determination of the HER2 amplification status by in situ fluorescent hybridization and concordance with immunohistochemistry for breast cancer samples in Colombia | 2013 | Cross-sectional | Y | Y | Y | Y | N | N | Y | Y | INCLUDE |
| HER2 testing in breast carcinoma: Very low concordance rate between reference and local laboratories in Brazil | 2011 | Cross-sectional | Y | Y | Y | Y | N | N | Y | Y | INCLUDE |

**Supplementary table 4** Quality assessment of the cross-sectional (a), and cohort studies included (b).
